# Empirical studies on informal patient payments for health care services: a systematic and critical review of research methods and instruments

**DOI:** 10.1186/1472-6963-10-273

**Published:** 2010-09-19

**Authors:** Tetiana Stepurko, Milena Pavlova, Irena Gryga, Wim Groot

**Affiliations:** 1 School of Public Health; National University of 'Kyiv-Mohyla Academy'; Ukraine; 2 Department of Health Organisation, Policy and Economics; CAPHRI; Maastricht University Medical Center; Faculty of Health, Medicine and Life Sciences; Maastricht University; The Netherlands; 3 Topinstitute Evidence-Based Education Research (TIER); Maastricht University; The Netherlands

## Abstract

**Background:**

Empirical evidence demonstrates that informal patient payments are an important feature of many health care systems. However, the study of these payments is a challenging task because of their potentially illegal and sensitive nature. The aim of this paper is to provide a systematic review and analysis of key methodological difficulties in measuring informal patient payments.

**Methods:**

The systematic review was based on the following eligibility criteria: English language publications that reported on empirical studies measuring informal patient payments. There were no limitations with regard to the year of publication. The content of the publications was analysed qualitatively and the results were organised in the form of tables. Data sources were Econlit, Econpapers, Medline, PubMed, ScienceDirect, SocINDEX.

**Results:**

Informal payments for health care services are most often investigated in studies involving patients or the general public, but providers and officials are also sample units in some studies. The majority of the studies apply a single mode of data collection that involves either face-to-face interviews or group discussions.

One of the main methodological difficulties reported in the publication concerns the inability of some respondents to distinguish between official and unofficial payments. Another complication is associated with the refusal of some respondents to answer questions on informal patient payments.

We do not exclude the possibility that we have missed studies that reported in non-English language journals as well as very recent studies that are not yet published.

**Conclusions:**

Given the recent evidence from research on survey methods, a self-administrated questionnaire during a face-to-face interview could be a suitable mode of collecting sensitive data, such as data on informal patient payments.

## Background

Informal patient payments are an important feature of health care systems in many countries around the world [1,2]. In some countries, like Kazakhstan, these payments even represent a significant part of the income of health care providers [3]. Various authors have explained their existence by cultural perceptions, insufficient funding of the health care sector and lack of control and accountability in the health care system [e.g. [2,4,5]].

Despite the different explanations, informal patient payments are overall seen as a negative feature of health care provision. Informal patient payments can have adverse effects on equity and can hinder the determination of future funding requirements of the health care sector [2,6-8]. Empirical studies on informal patient payments provide evidence on the scope and scale of this phenomenon. Such information could compel and enable policy-makers to look for solutions to the problem of informal patient payments [9]. This is particularly relevant to countries where informal patient payments are condoned by the government mainly because they are filling gaps caused by insufficient health care budgets [7,10,11].

Although the importance of data on informal patient payments is universally recognised, the collection of such data is a challenging task given their informal and potentially sensitive nature [12-14]. Moreover, Dabalen and Wane [15] state that informal patient payments are a sensitive research topic due to their illegal character in some countries. This implies difficulties in estimating their real scope and magnitude, and above all difficulties in determining the frequency of their occurrence. Furthermore, verification and validation of the estimates on informal payments are usually difficult. To assure validity and reliability, empirical studies on informal patient payments need to pay special attention on the research design applied [3].

Methodological difficulties in collecting sensitive data (as well as data that indicate potentially illegal behaviour) are an important topic in research on survey methods [e.g. [16-19]]. In this area of research, a sensitive topic is defined as a topic that "seems to be threatening in some way to those being studied" [20]. Empirical research on a sensitive topic requires special attention on two main issues: the development of an adequate research instrument as well as an adequate data collection process [e.g. [21,22]]. To respond to these methodological challenges, the empirical studies on informal patient payments have employed a variety of solutions. Thus, the studies considerably differ with regard to the methodology used. These differences may well affect the results of these studies and this creates difficulties in comparing their outcomes [e.g. [23]].

The aim of this paper is to critically review the research designs applied to the investigation of informal patient payments following the method of a systematic literature review. Our review is expected to facilitate the development of future research designs for collecting valid and reliable data on informal patient payments. Such critical review has not yet been reported in the literature. To achieve our aim, we first define the term "informal patient payments". Based on this definition, we identify keywords to search systematically for relevant publications. The following sections present our definition of informal patient payments and the methods of data collection, followed by the results and their discussion.

### Definition of informal patient payments

Empirical studies on informal patient payments attribute different characteristics to this type of payments. As a result, informal patient payments do not have a universal definition although the definitions used by researchers partly overlap.

For example, Adam [24] who reported on one of the first analyses on informal patient payments, uses the term "gratuity for doctors" referring to "a financial or other material benefit, given to the doctor voluntary by a patient or his/her relatives after the treatment has been terminated". More recent studies provide a broader definition of informal patient payments. Lewis [7] defines these payments as "payments to individual and institutional providers, in kind or in cash, that are made outside official payment channels or are purchases meant to be covered by the health care system". This definition includes "envelope payments to physicians" and "contributions to hospitals", as well as payments for medical supplies and pharmaceuticals purchased by the patient privately but intended to be covered by the government-financed health care system. Thompson and Witter [2] also provide a broad definition of informal patient payments but adding other dimensions: "tips for health workers", "bribes to obtain access to certain services or better quality care", and "payments demanded by health workers or institutions". They also refer to informal patient payments as payments that "are not sanctioned by the authorities".

Other recent studies add the moment of payment to the definition of informal patient payments. According to Allin et al [25] "informal payments range from the ex ante cash payment to the ex post gift-in-kind". The authors also outline some synonyms of the term "informal patient payments", including "under-the-table payments"' or "envelope payments". Other synonyms used, include "unofficial out-of-pocket payments", "under-the-counter payments" and "corruption in health care" [e.g. [6,26-28]]. However, corruption in health care has a wider meaning and includes not only informal patient payments, but also informal (illegal) payments to physicians and/or officials initiated by pharmaceutical companies (or other actors) for own, mainly financial benefits. Allin et al [25] as well as Balabanova and McKee [4] define patient payments as direct payments by patients for services that should in principle be provided free-of-charge and usually within the public health care system.

Despite the difference in the definitions, it is generally accepted that informal patient payments could have monetary and non-monetary form, and could express the patient's gratitude but could also be requested by the health care provider. Overall, informal patient payments are accepted to be unofficial, i.e. they are not registered by the state and are made without an official receipt of payment, and remain outside the official payment channels. However, confusion arises when these payments are also defined as illegal. This is because informal payments are sometimes - but not always - illegal [7]. These payments are not illegal as long as the existing laws and regulations are not contravened. Moreover, if informal payments - like gifts and donations - are not directly related to the treatment received, they are usually legal and frequently even tax deductable. It is also possible to define quasi-official payments, which include those payments that are illegal but for some reason tolerated by the government [7,10,11].

Based on the definitions of informal patient payments discussed above and reported in other publications [e.g. [29,30]], it is possible to define several key characteristics of informal patient payments, which can provide a base for a universal definition. These key characteristics include:

▪ Who initiates the informal payment? The patient who wishes to express gratitude, the provider (individual or institution) who requests the payment, or both?

▪ What is the nature of informal payment? In cash, in kind (e.g. candies, jewellery), or in a form of services (e.g. dinners, trips, and sponsorship)?

▪ What is the moment of informal payment? Before, during, or after the health care service, medical supplies or pharmaceuticals are provided to the patient?

▪ Who receives the informal payment? The health care institution (incl. quasi-official payments that are not official but when the patient receives a kind of receipt), medical staff (incl. physicians and nurses), or the administration of the health care institution?

▪ Who actually makes the informal payment? The patient or the relatives of the patient?

▪ What is the purpose of informal payment? Expression-of-gratitude, fee-for-service, fee-for-commodity, fee-for-access, fee-for-quicker-access, or fee-for-better-quality?

▪ What is the amount of the informal payment? The monetary value of the informal patient payment is usually compared to the household's income.

▪ How is the informal payment perceived? Normal behaviour, corruption, illegal behaviour, or tradition (due to cultural perceptions)?

▪ What is the attitude toward the informal payment? Negative (especially, if requested) or positive (if an expression of gratuity), usually depending on the moment of payment?

The characteristics of informal patient payments presented above, cannot be analysed separately because they may correlate among each other or one characteristic may even be a cause of another characteristic. For example, when the payment is requested, it is usually observed as a cash payment to medical staff in a surgical department, and the amount of the payment can be higher than the monthly income of the patient. At the same time, a gratuity payment that is in kind has a value that corresponds to the patient's income. Despite the possibility of such correlations, to be able to understand the phenomenon of informal patient payments, all characteristics listed above should be taken into account.

We take these characteristics as a definition of informal patient payments for our analysis. However, we focus solely on informal patient payments for health care services excluding informal patient payments for medical supplies and pharmaceuticals.

## Methods

In order to indentify the research techniques used in the study on informal patient payments for health care services, we conducted a systematic literature review using the method of desk research. The combinations of keywords used for the search of relevant literature, consisted of two components. The first component contained the term "informal patient payments" or one of its synonyms (see previous section), namely "unofficial out-of-pocket payments", "under-the-counter payments", "under-the-table payments", "envelop payments", and "corruption in health care". The second component consisted of the term "empirical research" or one of its synonyms, namely "survey" and "study".

Using all possible combinations of keywords in each of the two components, the following databases were searched: Econlit, Econpapers, Medline, PubMed, ScienceDirect, SocINDEX. Only English language publications were selected for further analysis. There were no limitations with regard to the year of publication or publication status. Each publication identified in the systematic search for literature, was checked for its relevance with regard to our research questions. Only publications that reported on empirical studies were included in the list of relevant publications. If it was obvious that the same empirical study was reported in more than one publication, only one publication was included in the final list but all publications that reported the study, were taken into account to identify details related to the study design. We also reviewed the reference lists of the publications that we identified for other relevant studies.

The content of the publications was analysed qualitatively and the results were organised in the form of tables. The main objective of the analysis was to outline the research designs reported in these publications. The focus was to extract information on the data collection process (i.e. sample characteristics and data collection mode) and the research instrument (i.e. groups of questions, pilot and pre-test, cross-national specificity and recall period). The research results reported in the studies were also summarised (using the definition outlined in the previous section) to indicate the type and incidents of informal payments for health care services reported in the literature. The results reported in the tables, were assessed in view of findings reported in the literature on research methods.

## Results

In total, 31 publications were identified as relevant in the systematic literature review. The publications are presented in Appendix 1 according to the year of their publication starting from the most recent ones. This section presents the general study description, data collection process, research instruments, as well as types and incidents of informal patient payments reported in these publications.

### General description of the publication

The general description of the studies included in our review is presented in Table 1. As indicated in Table 1, we identified 24 articles and 7 reports/books relevant to our analysis. Most of the field work reported in these publications had taken place over the period 1990 - 2005, while the publications date from 1995 to present. None of the papers contains an analysis of data collected before 1990. We observed that the number of publications was continuously growing: specifically, 7 publications appeared in the period 1995 - 2000 and a twice higher number appeared after 2005.

**Table 1:** General description of publications included in the analysis (31 publications reviewed)^1^

Classification category	Sub-categories	N	Reference index in Appendix 1
Type of publication	Journal articles	24	1,2,3,4,5,7,8,10,11,12,13,14,15,16,17,18,20,25,26,27,28,29, 30,31
	Reports, books	7	6,9,19,21,22,23,24

Year of publication	After 2005	13	1,2,3,4,5,6,7,8,9,10,11,12,13
	2001-2005	11	14,15,16,17,18,19,20,21,22,23,24
	1995-2000	7	25,26,27,28,29,30,31

Year of data collection	After 2005	1	6
	2001-2005	12	1,2,5,8,9,10,11,13,15,16,17,24
	1996-2000	9	12,14,18,19,20,21,22,25,26
	1990-1995	4	27,29,30,31
	Not clear	5	3,4,7,23,28

Origin of the study (type of country by World Bank)	Low-income countries	8	5,6,9,11,16,18,26,28
	Lower-middle-income countries	10	7,8,9,13,14,15,16,17,22,25
	Upper-middle-income countries	12	1,2,10,16,19,20,21,23,25,27,29,30
	High-income countries	9	2,3,4,12,16,21,24,25,31

Number of countries included in the study	Single country	26	1,3,4,5,6,7,8,10,11,12,13,14,15,17,18,19,20,22,23,24,26,27, 28,29,30,31
	Several countries	5	2,9,16,21,25

	Descriptive	15	8,10,11,13,15,19,20,21,22,23,24,25,27,28,31
	Analytical	18	1,3,4,5,12,13,14,15,16,17,18,19,20,23,24,26,29,30
Objective of the study	Predictive	3	7,13,19
	Not stated explicitly	3	2,6,9

^1^One publication can be associated with more than one sub-category.

Research on informal payments for health care services was conducted in virtually all continents. This includes countries with low-, lower-middle-, upper-middle- and high-income economies (country classification by World Bank, 2009 [31]). Moreover, we observed that the phenomenon was reported mostly in former-socialist countries but also in some countries that had not been socialist (e.g. Peru, Uganda, Greece and Turkey). From all 31 publications, only 5 publications reported cross-national studies. The rest of the studies reported results from a single country. One third of the studies had a descriptive aim and half of the studies had an analytical aim. Only in three cases, the research objectives could be described as predictive. None of the studies (even earlier studies) could be classified as having an exploratory aim.

### Specificity of the data collection process

The specificities of the data collection process reported in the publications that we reviewed, are presented in Table 2. We found out that the topic of informal payments for health care services was analysed from the perspective of members of households and patients, as well as from the perspective of health care providers and officials. The combination of several sampling units was also reported. The sampling area varied greatly: from a city and district to a single country and even several countries. Mostly, studies reported probabilistic sample designs (e.g. random, stratified or stratified random sample), although researches operated also with snowball and convenience samples. In total, 9 out of 31 publications had a sample size of less than thousand respondents and 12 out of 31 publications reported thousand to three thousands respondents.

**Table 2:** Specificities of data collection (31 publications reviewed)^1^

Classification category	Sub-categories	N	Reference index in Appendix 1
Sampling unit	General public: households	13	1,2,3,4,9,10,11,15,18,21,22,29,30
	General public: individuals	10	7,12,13,14,16,20,21,24,25,27
	Patients	5	5,17,23,26,28
	Providers	10	2,6,13,17,19,20,23,25,28,31
	Officials	3	2,19,24
	Other (newspapers)	1	8

Sampling area	Cities	9	1,7,10,14,21,22,23,27,30
	Districts	7	5,13,15,17,19,26,28
	Single country non-representative	7	3,4,8,24,25,29,31
	Single country representative	7	2,6,11,12,16,18,20
	Multiple country non-representative	4	2,9,21,25
	Multiple country representative	1	16

Sample selection	Random sample	8	4,12,13,15,20,21,27,30
	Stratified random sample	9	2,3,10,16,22,25,26,29,31
	Stratified sample	5	1,5,6,14,24
	Purposive sample	1	8
	Convenience sample	3	7,13,17
	Snowball sample	2	17,23
	Not presented	5	9,11,18,19,28

Sample size (units)	Higher than 10000	3	2,9,16
	2000 - 3000	5	11,14,18,24,29
	1000 - 2000	9	3,4,5,6,12,20,26,30,31
	Less than 1000	12	1,7,8,10,13,17,19,22,23,26,27,28
	Not presented	2	15,21

Number of data collection modes applied in the study	One type	18	1,3,5,6,7,8,10,11,12,16,19,21,22,24,26,27,30,31
	Two types	3	13,17,23
	Three types	3	2,20,25
	More than three types	1	28
	Not clear	6	4,9,14,15,18,29

Data collection mode applied for general public and patients	Self-administrated questionnaire	3	7,11,24
	Face-to-face structured interview	13	1,2,5,10,12,16,20,22,25,26,27,28,30
	Telephone interview	3	3,4,21
	Semi-structured/in-depth interview	5	13,17,20,23,25
	Focus-group discussion	6	2,13,17,23,25,28
	Not clear (interview/questionnaire)	5	9,14,15,18,29

Data collection mode applied for providers and officials	Self-administrated questionnaire	2	24,31
	Interview	8	6,13,17,19,20,23,25,28
	Focus-group discussion	5	2,13,23,26,28
	Stakeholder workshop	1	2
	Diary	1	28

^1^One publication can be associated with more than one sub-category.

Of all studies included in our review, 18 publications reported one type of data collection mode. In the case of consumers, the most frequently used mode of data collection was face-to-face interview. In the case of providers, face-to-face interviews were also widely used, but besides them, focus-groups interviews and self-administrated questionnaires were also used to gather data. Focus-group discussions and questionnaires were applied to consumers as well. Overall, self-administrated questionnaires were seldom used as a research instrument. Few publications reported a mixing mode of data collection combining interviews and group discussions. Respondents were not the only source of data. For example, one article provided content-analysis of printed media.

Additional analysis suggested that the collection of data on informal patient payments had changed over the years. At the beginning, only the general public and providers were involved in the studies, while later, patients and official were also included as sampling units. The number of sampling areas and sample selection techniques applied in the studies increased over the years. Thus, researchers included not only probabilistic sample designs but also purposive, snowball and convenience samples in the recent years, as well as larger sampling areas. The samples in some recent studies were very large (more than 10000 units) compared to earlier studies. The data collection had also become more varied with the years including more types of data collection modes. An interesting example is the stakeholder workshop applied in one recent study.

We also considered the response rate reported in the publications but we found that only 9 out of 31 publications indicated this feature of data collection. When reported, the response rate was quite high ranging form 70% to higher than 90%. Only one study based on telephone interviews reported a response rate lower than 20%. Nevertheless, the limited number of publications that report the response rate precludes a meaningful comparison in this direction.

### Specificity of the research instrument

Table 3 presents the specificity of the research instrument applied in the 31 studies that we reviewed. We divided the questions on informal payments for health care services described in the publications into questions to consumers (i.e. the general public and patients) and questions to the providers and officials. Thereby, in 19 studies, consumers were asked to estimate the size of informal patients for health care services. In 20 publications they recalled incidents of such payments. Moreover, the type of informal payments, beneficiary of these payments, reasons for making informal payments, perceived effects of payments and attitudes toward the presence of informal payments were also investigated. With regard to the officials and providers, other types of questions besides those for consumers were included, namely: reasons for receiving informal payments, mechanisms of collecting informal payments from patients, and methods of reducing the unofficial payments.

**Table 3:** Specificities of research instruments (31 publications reviewed)^1^

Classification category	Sub-categories	N	Reference index in Appendix 1
Groups of questions on informal patient payments for general public and patients	Incidents of informal payments	20	1,2,3,7,9,10,12,15,16,17,18,20,21,22,23,24,26,27, 29,30
	Types of informal payments	14	1,2,3,10,11,15,17,18,20,21,23,24,26,27
	Beneficiary of informal payments	16	1,2,3,10,11,12,15,17,18,20,21,23,24,26,29,30
	Moment of informal payments	4	2,10,17,20
	Magnitude of informal payments	19	1,2,3,4,5,9,10,12,14,15,16,18,20,21,22,23,24,29,30
	Reasons for informal payments	10	2,3,10,13,17,20,21,23,24,28
	Perceived effect of informal payments	3	2,13,20
	Attitudes towards informal payments	7	2,7,10,20,21,22,23

Groups of questions on informal patient payments for providers and officials	Incidents of informal payments	2	24,25
	Types of informal payments	4	20,24,25,31
	Moment of informal payments	1	20
	Frequency of informal payments	1	31
	Magnitude of informal payments	2	6,31
	Reasons for informal payments	6	13,19,20,23,24,25
	Attitudes toward informal payments	6	17,19,20,23,24,25
	Perceived effect of informal payments	1	13
	Mechanism of informal payments	3	17,19,24
	Reduction of informal payments	1	13

Pilot and pre-tests of the research instrument	Pilot study	10	1,7,13,16,20,21,25,26,28,30
	Pre-test	2	7,17
	Not presented	19	2,3,4,5,6,9,10,11,12,14,15,18,19,22,23,24,27,29,31

Cross-national specificity of the research instrument	Backward translation	3	2,16,25
	Country specific part	2	2,16
	Not presented	2	9,21

Recall period of the experience or the general public and the patients	Less than 1 month	1	18
	1-5 months	9	1,2,5,7,9,11,14,15,17
	6-11 months	5	15,18,20,26,27
	12 months and more	11	3,4,5,9,11,12,16,18,22,29,30
	Other (last visit, 3 last visits)	3	2,7,24
	No recall period (next visit)	2	7,20
	Not clear	6	10,13,21,23,25,28

Recall period of the experience for providers and officials	Previous week	1	31
	2 years	1	25
	Not clear or not applicable	9	6,13,17,19,20,23,24,26,28

^1^One publication can be associated with more than one sub-category.

Although, we could identify groups of questions on informal payments for health care services included in the studies, overall, the content of the research instrument was rarely described in detail. The piloting and pre-testing were also not described in details in any of the publications.

With regard to the recall period, researchers frequently appealed to the memory of respondents when the experience with paying informally was the objective of the survey. There were only two options of the recall period applied to the providers and officials: last week and two years ago. However, we found a variety of recall periods applied in studies among consumers. Respondents were asked to remember making payments during a year or more, as well as during one to five months. Next visit and last visit were also used as reference points in the studies.

Two publications stated that the questionnaire was pre-tested and 10 publications provided information that the questionnaire was piloted. In cross-national studies, a backward translation was usually applied to ensure the proper wording of the questions. The introduction of country specific questions was also used in these studies.

### Description of the main findings

Tables 4 and 5 contain the key empirical findings presented in the publication with regard to the type and incidents of informal payments for health care services. The findings are presented systematically in the tables based on our definition of informal patient payments outlined at the outset of this paper. Both patients and providers were reported as the ones that initiate the informal payments for health care services. Informal payments in cash and in kind were equally reported. However, some early publications reported also informal patient payments in a form of service, e.g. car repairs, plumbing, sponsorship for conference participation. Informal patient payments that were in cash, were mainly paid before or during the treatment and gifts were mainly presented after the service was provided.

**Table 4:** Types of informal payments reported (31 publications reviewed)^1^

Classification category	Sub-categories	N	Reference index in Appendix 1
Who initiates the informal payment?	Patients (expression of gratitude)	9	11,15,17,20,21,22,23,24,25
	Provider (demanded by a provider)	9	3,11,17,20,21,22,23,24,25

What is the nature of informal payment?	Payments in cash	20	1,2,3,5,10,11,15,16,17,18,20,21,22,23,24,25,27,29,30,31
	Payments in kind (gifts)	18	1,2,3,5,10,11,15,16,17,18,20,21,23,24,25,27,29,31
	Payments in a form of services	4	17,20,24,31

What is the moment of informal payment?	Before/during treatment (mostly in cash)	5	2,10,17,20,23
	After treatment (mostly gifts)	3	2,20,23

Who receives the informal payment?	General practitioner	10	2,5,11,12,17,21,23,24,27,30
	Medical specialist	6	2,3,6,12,21,24
	e.g. Surgeons	7	1,10,11,19,20,21,23
	e.g. Dentists	4	21,24,29,30
	e.g. Obstetrics-gynaecologist	4	11,19,20,23
	Other medical staff	3	3,11,30
	e.g. Nurses	6	3,6,17,21,25,30
	e.g. Emergency staff	1	24
	Health care institution (Incl. quasi-official payments when the patient receives a kind of receipt)	3	10,25,29

What is the purpose of the informal payment?	Expression-of-gratitude	10	1,2,8,13,17,20,21,22,24,25
	Fee-for-service	6	13,20,21,22,23,27
	Fee-for-commodity	4	17,21,23,27
	Fee-for-access	4	8,13,17,27
	Fee-for-quick-access	6	2,3,13,17,23,24
	Fee-for-better-quality	10	1,2,10,13,17,20,21,22,23,24
	Fee-for-psychological-comfort	4	3,13,20,28

What is the amount of informal payment? (% of monthly income)	Less than 30%	3	2,20,30
	More than 80%	2	17,30

How is the informal payment perceived?	Tradition/gratitude	4	3,17,20,23
	Illegal behaviour	1	22
	Corruption	3	2,20,22

What is the attitude of the respondent toward the informal payment?	Negative (requested)	6	7,10,19,20,21,23
	Positive (gratuity)	5	7,19,20,21,23

^1 ^One publication can be associated with more than one sub-category.

**Table 5:** Incidents of informal payments reported (31 publications reviewed)^1^

% respondents	Informal payments in general	Gifts or gratuities only	Cash payments or extra fee only	Reference index in Appendix 1
		
	N	N	N	
1-10%	3	1	3	2,9,11,21,24,26,27
11-20%	4	2	1	2,3,9,12,18,20,21,27
21-30%	2	3	2	1,10,15,18,20,24
31-40%	4	-	-	3,7,10,16,
41-50%	1	1	2	18,24,29,30
51-60%	2	-	-	12,24
61-70%	2	-	1	1,7,15
More than 71%	1	2	-	7,20

^1^One publication can be associated with more than one sub-category.

Researchers reported a variety of beneficiaries of informal payments for health care services, e.g. general practitioners, medical specialists, other medical staff, and administration. Overall, respondents reported higher informal payments for services of medical specialists (notably surgeon and dentist) than for services of general practitioner although we observed that researchers appealed more often to informal payments to general practitioners. The expression of gratitude was identified as a motivation for informal patient payments in about a quarter of the studies while more than a quarter of the studies reported the improved service provision (better quality and quicker access) as the main reason for such payments.

The magnitude of informal patient payments was rarely reported (only in 5 publications). Nevertheless, this characteristic of informal patient payments was hardly comparable since researchers were using different measurement units: monthly household income, or monthly household expenditure, or health expenditures.

The few studies that investigated the perception and attitude of respondents toward informal patient payments, reported quite contrasting results. Informal patient payments were perceived by respondents as tradition and gratuity in 3 studies, and in other 3 studies they were perceived as illegal behaviour and corruption.

The incidence of informal patient payments reported in the publications that we reviewed (see Table 5), differed significantly. Although, the studies often offered the percentage of respondents that had made informal payments for health care services, this percentage was estimated in different manners: percentage of all respondents, percentage of health care consumers, or percentage of patients who paid for the treatments. This precludes the possibilities for further conclusions based on Table 5.

### Methodological difficulties and limitations reported in publications

The publications included in our review, reported and discussed methodological difficulties and limitations. One of the main research problems concerns the respondents' understanding of the concept informal payments. In particular, respondents were often unable to distinguish between official and unofficial payments, which made the estimation of the magnitude of informal patient payments very approximate [14,15,27]. Another problem related to data validity, was the refusal of some respondents to answer questions on informal patient payments when filling in a questionnaire or asked by interviewer [32]. Nevertheless, Belli et al [33] provide evidence that users' and providers' answers were frank and open, and Barr [34] observed that providers did not look confused while filling in the questionnaires. Some researchers [e.g. [23]] indicated possible uncertainty about the accuracy of responses to questions on informal patient payments when an interviewer was present. Methodological limitations such as sample design, units of analysis applied, memory recall bias, and under- or over-estimation of the informal payments were also stated by the authors.

## Discussion and conclusions

Informal patient payments are a multi-face phenomenon with different features even within a single country (i.e. in the frame of the same health care system, regulations and traditions). Therefore, a universal definition is not available. The key characteristics described at the outset of this paper provide a more appropriate base for studying this phenomenon than pursuing an all-inclusive definition. Still, country-specific features should be taken into account to make sure that the unit used to measure informal payments is meaningful to the population being sampled.

The results of our review suggest that the study of informal patient payments for health care services is rather new, though the phenomenon has been in existence for a number of decades [24]. Most of the studies that we identified were conducted between 1990 and 2005 mostly in former-socialist countries. It is likely that during the communist period, it was not possible to collect and report data on informal patient payments in these countries. Ideology also made it difficult to discuss the issue openly. Moreover, these types of payments might have been perceived as illegal. With the end of the communist governing, the socio-political changes resulted in more public attention to social problems, such as informal payments for health care services, which motivated their investigation. In addition, data collected since 2005 might still be in the stage of data analysis and therefore, not yet published. Overall, the dynamics of publications on informal payments indicates the growing research interest in this topic including new research techniques and larger sampling areas.

Our findings confirm that informal payments exist in countries of all levels of economic development, and in different parts of the world. However, we did not find studies reporting informal patient payments in high-income countries in North-West Europe, North America and Australia. The phenomenon is most often observed in former-socialist countries and developing countries (in Africa, South America and Asia), although it also exists in some high-income European countries that were not former-socialist countries (Italy, Greece, and Turkey) [35,36]. As mentioned at the outset of this paper, the literature offers various explanations why informal patient payments exist in these countries. This includes under-funding of the health care systems, the specific organisation and governance of the health care sectors, but also culture and social perceptions [33]. Still, these are only hypothesis and they need to be tested to explain the existence of informal patient payments in some parts of the world and their absence in others.

When we look at the study designs that we reviewed, we can outline several discussion points relevant to research. The first discussion point refers to the study objectives. We differentiated between exploratory, descriptive, analytical and predictive aims. However, we did not find studies with an explicit exploratory aim even among the earlier studies. We expected that an exploratory aim would be typical for the early studies when scant information was available because then, the research interest would be concentrated on exploring the phenomenon. Although some earlier studies had an explicit descriptive aim, other earlier studies had an analytical aim. Descriptive and analytical objectives allow finding determinants of informal patient payments and their correlation.

The second discussion point refers to the sample design. Sample design is part of the entire research design and it may minimise some biases in case of a well-developed sample. To estimate the level of informal patient payments, a probabilistic sample strategy is commonly implemented as it gives equal chances of being included in the study. Moreover, triangulation of the data is feasible when all parties participate (e.g. consumers, providers, officials) in the research. For instance, Cockroft et al [27] present quantitative data collected from households, where the main findings are discussed with physicians and nurses, as well as with stakeholders, to define the policy implications of the results. However, in the studies that we reviewed, the sample is not always constructed to avoid biases and to get valid data for the analysis. More pragmatic reasons, such as available research funds, are also reported [37,38]. In view of this, it is not surprising that some recent studies on informal patient payments applied purposive, snowball and convenience samples.

Another discussion point is the data collection mode. The mode of data collection can be especially problematic when sensitive data are studied. This is because each single mode of data collection has its own pros and cons when sensitive questions are asked. The mode of data collection might even be a determinant of the value of indictors estimated based on sensitive data [e.g. [17,19]]. According to our results, the response rate (when reported) was highest in face-to-face interviews with both consumers and providers. Face-to-face interviews are considered the most adequate approach in gaining understanding of what respondents mean when answering questions [39]. However, face-to-face interviews might not be very effective in assuring the validity of the data when such sensitive topic as informal patient payments, is addressed. Respondents might be less willing to reply truthfully to questions on illegal expenditures if asked by an interviewer since the level of confidentiality is lower. In contrast, self-completion methods are usually preferred when the subject matter is sensitive [37,40] even though some questions might be left unanswered by the respondents. The issue of confidentiality plays a key role. Respondents may be unwilling to describe their informal payments in front of an interviewer, and may feel more comfortable to express such behaviour when the pen in hand is the only "eyewitness". To overcome this difficulty, mixing modes of data collection could be used. De Leeuw [38] gives an example of U.S. National Survey on Drug Use and Health where respondents use a computer for answering sensitive questions while several non-sensitive questions are asked by an interviewer. This could also increase the response rate [41]. Evidence from research on survey methods confirms the importance of combining various modes of data collection in surveys where potentially sensitive issues are investigated. The objective should be to help respondents to exert the necessary cognitive efforts and to answer the questions carefully. At the same time, the objective should be to make the respondents comfortable enough to answer openly and honestly the questions that might be of a sensitive nature [18]. Thus, using mixing modes of data collection, specifically the introduction of a self-administrated part during a face-to-face interview, could be suitable for collecting valid data on informal patient payments. To the best of our knowledge, such a mixing mode of data collection has not been used in studies on informal patient payments.

Virtually all publications that we reviewed are based on retrospective research, thus another relevant discussion point is the recall period. The human memory can be a source of bias in research [39]. Consumers might not remember the exact number of visits to health care providers/facilities if the recall period is long (e.g. one or two years), all the more so, the amount of payments they have done. Overall, respondents remember the event for a longer period of time if it is important to them [37]. Thus, the experiences of utilisation of health care services can be different in case of less severe health complications (e.g. out-patient visits) and more severe health problems (e.g. in-patient services). Therefore, we recommend introducing different recall periods for questions on out-patient and in-patient services could enable the respondents to make less cognitive efforts. In particular, Baschieri and Falkingham [42] apply a 30 days recall period for utilisation of health care services and expenditures associated with visits to physicians and one year period for hospitalisations. There is also a possibility to avoid the use of a recall period. For example, the researcher's choice may lay on the introduction of diaries, which could allow collecting all expenditures of the household on health care at the time of payment. The choice of an adequate recall period is especially important for the valid measurement of informal patient payments.

Our findings on the response rate were surprising to a certain extent. In general, the response rate reported in the publications that we reviewed, was rather high. This could suggest that people are willing to talk about informal patient payments despite their informal and potentially illegal nature. However, it should be recognised that only few publications presented this characteristic. It might be that the response rate was presented in these publications because it was favourable for the study and indicated the representativeness of the data.

To enrich the methodological approaches to the investigation of informal patient payments, researchers can appeal to methods for measuring corruption in society. Although informal patients are not always illegal, our review suggests that they are sometimes perceived by respondents as corruption and illegal behaviour. The literature on measuring corruption suggests that corruption can be studied through the measurement of perceived corruption, as well as perceived willingness to pay bribes and bribe payments [43]. Specifically, studies that focus on corruption include questions on the respondents' perception about level of corruption in a country, as well as hypothetical questions about the amount of money that a respondent would be willing to pay as a bribe in a given context. The latter technique could be useful to study respondents' attitude toward corruption. The measurement of both perceived corruption and willingness to pay bribes and bribe payments could be especially appealing for the investigation on informal patient payments to gain a better understanding on why informal patient payments exist.

The key results on the type of informal patient payments indicate that informal patient payments are a multifaceted phenomenon. All characteristics of informal patient payments included in our definition, appeared relevant for describing the pattern and magnitude of informal payments for health care services. Overall, the results indicate a great variety in the types of informal patient payments reported. This needs to be considered when designing a research instrument for the investigation of these payments. In particular, the researcher needs to clarify in advance what types of informal patient payments should be studied and thus, what type of questions to be included. It is also important to decide how to measure the incidents of informal patient payments since various measurement units are possible.

Our attempt to compare the empirical results presented a significant challenge. This is mainly due to the great variety of research methods applied. However, the overall findings indicate that informal patient payments are a substantial phenomenon in terms of both scope and scale, and should not be neglected. Moreover, results of household surveys would be more meaningful if considered against the background of macro-level data at a national level (whenever available). For example, the National Health Accounts could be a useful source of macro-level data since they report total health expenditures as well as formal transactions in the health care sector (e.g. expenditures by various institutions, external financing and out-of-pocket spending). In addition to this, little is known on why informal patient payments exist and how the specific patient-providers relationship determines them. This indicates the need of combining quantitative and qualitative research methods when studying this type of payments. The need of deeper understanding of the informal patient payments has already captured the attention of researchers who are trying to provide theoretical explanations to the existing empirical findings [5,44].

We searched systematically for relevant publications. However, we do not exclude the possibility that we have missed some studies reported in non-English language journals as well as very recent studies that are still not reported. Despite this shortcoming, our results and discussion are relevant to future research on informal patient payments. As mentioned above, the investigation of the phenomenon is interwoven with methodological complexities related primarily to the data collection and research instruments. We have outlined and discussed most of these complexities. However, other peculiarities (e.g. wording of the questions and the length of the interview) also require attention.

Based on our findings in combination with the conclusions of a recent methodological review presented in Roberts [18], the following key strategies could be recommended to researchers who choose to study informal patient payments: (1) considering a broad county-specific definition of informal patient payments when designing the questionnaire and an adequate measurement unit that is meaningful to the population being sampled; (2) opting for face-to-face interviews at the respondents' home to ensure that the interview situation is adequately conducive to respondents but simultaneously, to enable a high response rate; (3) administrating the questions on informal patient payments as an anonymous self-completion component within the face-to-face interview; (4) assuring the respondents on the issues of confidentiality and explaining why the data on informal patient payments are important.

## Appendix 1. Index of publications included in the review

1. Ozgen H, Sahin B, Belli P, Tatar M, Berman P: **Predictors of informal health payments: The example from Turkey**. *Journal of Medical Systems *2010, **34(3): **387-396.

2. Cockcroft A, Andersson N, Paredes-Solis S, Caldwell D, Mitchell S, Milne D, Merhi S, Roche M, Konceviciute E, Ledogar R: **An inter-country comparison of unofficial payments: Results of a health sector social audit in the Baltic States**. *BMC Health Services Research *2008, **8:**15.

3. Liaropoulos L, Siskou, O, Kaitelidou D, Theodorou M, Katostaras T: **Informal payments in public hospitals in Greece**. *Health Policy *2008, **87:**72-81.

4. Siskou O, Kaitelidou D, Papakonstantinou V, Liaropoulos L: **Private health expenditure in the Greek health care system: Where truth ends and the myth begins**. *Health Policy *2008, **88**:282-293.

5. Tediosi F, Aye R, Ibodova S, Thompson R, Wyss K: **Access to medicines and out of pocket payments for primary care: Evidence from family medicine users in rural Tajikistan**. *BMC Health Services Research *2008, **8**:109.

6. Dabalen A, Wane W: *Informal payments and moonlighting in Tajikistan's health sector*. Washington, D.C.: The World Bank and Development Research Group; 2008.

7. Burak LJ, Vian T: Examining and predicting under-the-table payments for health care in Albania: An application of the theory of planned behavior. *Journal of Applied Social Psychology *2007, 37: 1060-1076.

8. Chiu YC, Smith KC, Morlock L, Wissow L: **Gifts, bribes and solicitions: Print media and the social construction of informal payments to doctors in Taiwan**. *Social Science & Medicine *2007, **64:**521-530.

9. Hunt J: *Bribery in Health Care in Peru and Uganda*. NBER Working Paper. Cambridge: NBER; 2007. http://www.nber.org/papers/w13034

10. Tatar M, Ozgen H, Sahin B, Belli P, Berman P: **Informal payments in the health sector: A case study from Turkey**. *Health Affairs *2007, **26:**1029-1039.

11. Baschieri A, Falkingham J: Formalizing informal payments: The progress of health reform in Kyrgyzstan. *Central Asian Survey *2006, 25:441-460.

12. Szende A, Culyer AJ: **The inequity of informal payments for health care: The case of Hungary**. *Health Policy *2006, **75:**262-271.

13. Vian T, Grybosk K, Sinoimeri Z, Hall R: **Informal payments in government health facilities in Albania: Results of a qualitative study**. *Social Science & Medicine *2006, **62:**877-887.

14. Gotsadze G, Bennett S, Ranson K, Gzirishvili D: **Health care-seeking behaviour and out-of-pocket payments in Tbilisi, Georgia**. *Health Policy and Planning *2005, **20:**232-242.

15. Hotchkiss DR, Hutchinson PL, Malaj A, Berruti AA: **Out-of-pocket payments and utilization of health care services in Albania: Evidence from three districts**. *Health Policy *2005, **75:**18-39.

16. Balabanova D, McKee M, Pomerleau J, Rose R, Haerpfer C: **Health service utilization in the Former Soviet Union: Evidence from eight countries**. *Health Services Research *2004, **39:**1927-1950.

17. Belli P, Gotsadze G, Shahriari H: **Out-of-pocket and informal payments in health sector: Evidence from Georgia**. *Health Policy *2004, **70:**109-123.

18. Falkingham J: **Poverty, out-of-pocket payments and access to health care: Evidence from Tajikistan**. *Social Science & Medicine *2004, **58:**247-258.

19. Shishkin S, Bogatova T, Potapchik Y, Chernets V, Chirikova A, Shilova L: *Informal Out-of-Pocket Payments for Healthcare in Russia*. Independent Institute for Social Policy: Moscow; 2003.

20. Balabanova D, McKee M: Understanding informal payments for health care: The example of Bulgaria. *Health Policy *2002, 62:243-273.

21. Belli P: *Formal and informal household spending on health: a multi-country study in central and eastern Europe*. Cambridge, MA, Harvard School of Public Health; 2003. Belli, 2002.

22. Litvak A, Pogorilyi V, Tyschuk M: *Underground Economy in Health Care in Contemporary Ukraine*. Odesa: TEC; 2001.

23. Shahriari H, Belli P, Lewis M: *Institutional Issues in Informal Health Payments in Poland: Report on the Qualitative Part of the Study*. HNP Discussion Paper. Washington, D.C.: The World Bank; 2001.

24. Anderson J: *Corruption in Slovakia: Results of diagnostic surveys*. Washington, D.C.: The World Bank and USAID; 2000.

25. Miller WL., Grodeland, AB, Koshechkina TY: **'If you pay, we'll operate immediately'**. *Journal of Medical Ethics *2000, **26:**305-311.

26. Killingsworth JR: **Unofficial fees in Bangladesh: Price, equity and institutional issues**. *Health Policy and Planning *1999, **14:**152-163.

27. Mastilica M, Bozikov J: **Out-of-pocket payments for health care in Croatia: Implications for equity**. *Croatian Medical Journal *1999, **40**:152-159.

28. McPake B, Asiimwe D, Mwesigye F, Ofumbi M, Orteublad L, Stree P: **Informal economic activities of public health workers in Uganda: Implications for quality and accessibility of care**. *Social Science & Medicine *1999, **49**:849-865.

29. Chawla M, Berman P, Kawiorska D: **Financing health services in Poland: New evidence on private expenditures**. *Health Economics *1998, **7:**337-346.

30. Delcheva E, Balabanova D, McKee M: **Under-the-counter payments for health care: Evidence from Bulgaria**. *Health Policy *1997, **42:**89-100.

31. Barr DA: The ethics of Soviet medical practice: Behaviours and attitudes of physicians in Soviet Estonia. *Journal of Medical Ethics *1996, 22:33-40.

## Competing interests

The authors declare that they have no competing interests.

## Authors' contributions

TS carried out the searching of the literature, drafted the results tables and manuscripts; MP developed the design of the study and a conception for data interpretation; improved the draft; IG participated in the sequence alignment, revised the paper; WG participated in the sequence alignment; revised the paper. All authors read and approved the final manuscript.

## Pre-publication history

The pre-publication history for this paper can be accessed here:

http://www.biomedcentral.com/1472-6963/10/273/prepub
